# COVID-19 Vaccine Superstations as a Model to Rapidly Achieve Herd Immunity

**DOI:** 10.1017/ash.2021.101

**Published:** 2021-07-29

**Authors:** Jocelyn Keehner, Francesca Torriani, Shira Abeles, Lucy Horton

## Abstract

**Background:** The County of San Diego Health and Human Services (SDHHSA) established a goal to vaccinate 1.9 million residents as quickly as possible to attain vaccine induced herd immunity. This strategy would minimize the emergence of more transmissible variants, to which some vaccines may be less effective. With this strategy in mind, UC San Diego Health (UCSDH) collaborated with the local health authorities and the San Diego Padres to build a superstation in downtown San Diego in the parking lot of a baseball stadium. **Methods:** Building on the experience of rapidly vaccinating the UCSDH workforce in mid-December 2020, UCSDH and SDHHSA partnered to more efficiently distribute SARS-CoV-2 vaccine in San Diego County by building a vaccine superstation. The San Diego Padres offered their parking lot as the site; it was centrally located, easily accessible, quick to set up, and semipermanent. They also provided infrastructure support, event coordination, and internet capability. Occupying a space of ~6.5 acres, the superstation included 12 lanes serving 12 cars each, with ~3 cycles every hour, as well as a pedestrian walk-up station. Altogether, the site had the capacity for >5,000 vaccinations daily. This effort required coordination among administration, healthcare providers, IT specialists, and support staff—a daily workforce of >300 persons. The workforce needs were met using a multipronged approach, including flexible staffing, coordination of volunteers, and recruitment of previously retired providers. The private–public partnership enabled the superstation to be up and running in 5 days. **Results:** The operation was quickly ramped up to provide >6,000 vaccines daily. Initially only open to healthcare workers, on January 17 the superstation was expanded to persons aged >75 years, with further expansion to those aged ≥65 years on January 23. From January 11 to February 5, >100,000 individuals received their first dose of vaccine at the superstation, corresponding to ~31% of all San Diego county vaccinations. **Conclusions:** Vaccination of as many people as quickly as possible is essential to controlling the pandemic. Unchecked replication of SARS-CoV-2 allows increases the chance that the virus may develop mutations that render vaccines and therapeutics less effective. Our model vaccine superstation was replicated at 3 more sites around the county, the limited allocation of vaccine has been the only barrier to further expansion. A force of 10 superstations could administer a first dose to the remaining 1.6 million county residents within 32 days.

**Funding:** No

**Disclosures:** None

